# Expression of Plasmid Encoded GRA4 Gene of *Toxoplasma gondii* RH Strain in CHO Eukaryotic Cells

**Published:** 2018

**Authors:** Marjaneh AGHDASI, Fatemeh GHAFFARIFAR, Fatemeh FOROOGHI, Abdol Hossein DALIMI ASL, Zohre SHARIFI, Nahid MASPI

**Affiliations:** 1. Dept. of Parasitology, Faculty of Medical Sciences, University of Tarbiat Modarres, Tehran, Iran; 2. Research Center of Iranian Blood Transfusion Organization, Tehran, Iran; 3. Dept. of Parasitology, Faculty of Medicine, Ilam University of Medical Sciences, Ilam, Iran

**Keywords:** *Toxoplasma gondii*, GRA4 gene, Gene expression, CHO cell

## Abstract

**Background:**

Toxoplasmosis is a common infection all around the world. During pregnancy; it may lead to congenital disorders or abortion in human and animals. Severe damage of toxoplasmosis indicates to require effective vaccine. One of dense granules antigen is GRA4 that secrete from tachyzoite and bradyzoite. GRA4 genome is unique without intron and is one of the major immunogenic proteins from *Toxoplasma gondii.*

**Methods:**

We confirmed the cloning of GRA4 gene into pcDNA3 by restriction enzyme and PCR of GRA4 gene with pcGRA4 plasmids as template. Then with using calcium-phosphate method we transfected the pcGRA4 into CHO (Chinesehamster ovary) cells. The yielded protein was separated by SDS-PAGE and moved by electroblotting to nitrocellulose paper.

**Results:**

Result of SDS-PAGE analysis showed the appearance of band approximately 42 kDa which was absent in the negative control, that was able to identify toxoplasmosis antibody IgM^+^ serum in western blot analysis.

**Conclusion:**

pcGRA4 plasmid is able to synthesis of antigenic protein in CHO cells. The ability of pcGRA4 for induction of protective immune response against toxoplasmosis will be evaluated in mouse model.

## Introduction

*Toxoplasma gondii* is apicomplexan parasite with a worldwide distribution that can cause toxoplasmosis infection (1). In immunocompromised hosts such as AIDS patients, the infection is severe. Primary acquired infection during pregnancy can be transmitted to the fetus and can produce severe symptoms such as miscarriage, neurological damage, ocular complications and other defects (2).

Consumption of food contaminated with tissue cysts or ingestion of oocysts released in the feces of infected cats can transmit this infection to human (3). Although drugs for treatment of toxoplasmosis infection in the acute phase are the main strategy, drug-resistance and side effect is seen, and they do not have effect on the chronic phase of infection (4). Protection against infection is mediated by T cell and involves both CD4+ and CD8+ Tcells (5).

The only industrial vaccine is, attenuated tachyzoite S48 strain famous as Toxovax (6). Which has short shelf-life, unwanted effects and high cost (7). Therefore, a new and affordable recombinant vaccine which stimulates T-cell-mediated protective immunity is needed.

In both infections acute and chronic phase excreted/secreted antigens (ESA) of *T. gondii* play main function in the stimulation of the host immune system (8). GRA proteins localized in both the PV and the cyst wall, several GRA gene expression demonstrate that GRA proteins are important for maturation of PV and transformation into a cyst (9, 10).

One of dense granule antigen is GRA4 that secrete from tachyzoite and bradyzoite, GRA4 genome is unique without intron (11). Oral infection with *T. gondii* induces both humoral and cellular immune responses by GRA4 peptides because amino acids 229–242 and 231–245 are epitopes for B and T-cell (12, 13). Many researchers candidate the GRA4 for vaccine and immunization against *T. gondii*.

The effect of plasmid containing SAG and GRA4 genes was studied with and without plasmid GM-CSF. The first one increases toxoplasmosis resistance, while the second one has higher protective effect (14). Martin et al. focused on the GRA4 recombinant proteins and ROP2 *T. gondii* along with alum in mice C57BL/6 and C3H. GRA4 and GRA4-ROP2 plasmids revealed similar levels of IgG isotypes against GRA4, but immunization with both plasmids caused higher level of IgG1 against ROP2 (15). Zhang et al. combined recombinant expression plasmids and vaccina virus both of which contained GRA4, and injected the mice. The mice with lethal doses of *T. gondii* challenge remained alive. Cysts formation was blocked in mice immunized by the primary regime and heterologous reinforce (16).

In this article, we explain the expression of *T. gondii* GRA4 gene by pcGRA4 in CHO cells and confirm it by SDS-PAGE and Western blot analyses.

## Materials and Methods

### Confirming the GRA4 cloning in pcGRA4 recombinant expression plasmid with KpnI and EcoRI enzymes

We firstly extracted pcDNA3 and pcGRA4 plasmids existed in the transformed bacteria, grown in LB medium containing ampicillin (17). The extraction was performed using the plasmid extraction kit (made by Roch Germany Company). Simultaneous application of the two enzymes has been used for enzyme cutting in double digestion way, and the recombinant plasmid was cut concurrent with the expression plasmid pcDNA3 (as a control sample) using KpnI and EcoRI. According to the Fermentas company kit instruction, the enzyme reaction to the volume of 20 mL were placed at 37° C overnight after vortex and spine (it contained 5 mL pcGRA4 recombinant plasmid, 1 unit EcoRI enzyme, 1 unit KpnI enzyme, 2 mLtango buffer and 11 mL distilled water). The result of the enzyme cut along with a molecular weight marker was electrophoresed on agarose gel.

### GRA4 gene PCR by using pcGRA4 recombinant plasmid as a template

With using specific primers, we determine presence of the GRA4 gene in expression plasmid and separate the recombinant plasmid from other plasmids (18).

The forward and reverse primers were designed according to the nucleotide sequence in Gene Bank database (https://www.ncbi.nlm.nih.gov/genbank/) with accession No. EU660037 and 1058 bp and Gen Runer Software. The specific primers were as follows:

Forward primer: 5′-CGCGGGTACCATGCAGGG-CACTTGGTTTTC-3′
Reverse primer: 5′-CGCGGAATTCTCACTCTTTGCG-CATTCTTT-3′
EcoRI: GAATTC
KpnI: GGTACC

PCR reaction to the volume of 25 mL was performed:

10 × PCR buffer2/5μl, 50mM MgCl2 0/75 μl,10mM dNTP 0/5 μl,10 Pomol/μ1 primer forward 1μl,10Pmol/μl primer Reverse 1μl,(5u/μl)Taq DNA Polymerase 0/5 μl, Extracted DNA 3μl, ddH2O 15/75μl. The above materials were placed on a vial 0. 5ml, after vortex and spine. Then, they were placed in thermocycler and PCR was conducted based on the following plan: denaturation 60 sec at 94°C, annealing 30 sec at 60°C, extension 1 min at 72°C. These processes were repeated for 30 cycles and the PCR product was loaded on agarose gel and electrophoresed (18).

### Transfection of pcGRA4 recombinant plasmid into the CHO eukaryotic cells

CHO cells were used as pcGRA4 recombinant plasmid host to express GRA4 gene protein. Eukaryotic cell was cultured in flasks of 75 ml at 37 °C and 5% CO_2_. For each 100 ml DMEM medium, 10 ml of sterile FCS and 1 ml combination of antibiotics (penicillin 100 unit/ml, streptomycin 100 unit/ml) were added. The number of 1–3**×**10^6^ eukaryotic cells were cultured in each 35 mm well in six-cell plate. When the cells filled 50%–80% of the plate, transfection was performed using calcium phosphate method. Then, 100 mL of calcium chloride 2. 5 M has added to 25 **μg/ml** DNA plasmid diluted at a ratio of 1/10 with Tris-Hcl buffer. The volume was reached to 1ml using distilled water. One volume of this solution 2xCa/DNA was rapidly and suddenly added to the same volume of 2x HEPES solution (1. 5mm Na2HPO4, 140 mm NaCl, 50 mm HEPES, pH 7. 05 at 23 °C), thena slight smog appeared within a few seconds, which marks the formation of sediment. The solution was centrifuged in 30 sec and 16000 gr round (at 0 °C) and quickly 250 mL of the above solution was removed, the light absorption was measured against a blank without DNA and phosphate at a wavelength of 320 nm. Measurement of the light absorption is used to confirm DNA connection to the precipitate. For each 1 ml medium, 100 μl precipitate was added to plate containing the cell and the plates were incubated for 72 h. Then, a transfected well and a control well (plasmid without GRA4 gene) were collected in a completely sterilized condition. The cells were washed with sterilized PBS then 400 μl PBS was added to each well. The cells were separated from the plane’s floor. For this purpose, the contents of each well were passed through sampler and collected in a vial of 1. 5 ml to be kept at −20°C until the usage time (19).

### SDS PAGE and Westernblot

To confirm GRA4 gene expression in eukaryotic cell, we applied acrylamide gel 10% electrophoresis. Moreover, we used freeze and thaw method to break transfected and nontransfected cells. Four microliter antiprotease was added to vial (cell volume 0. 01) and the cells were frozen and thawed several times. Then, they were centrifuged for five minutes in 3000 rpm and the supernatant was examined using SDS-PAGE and Westernblot method. A volume of the sample buffer SDS-PAGE was mixed with five volume of the sample and boiled for five minutes. 50μl of samples were placed in wells and the container was connected to the power supply (120 v) and transferred the protein bands of acrylamide gel into a nitrocellulose paper, then blocking solution (1% BSA-PBST20) was added. And was kept at 4 °C overnight. Finally, paper was removed from blocking solution and put in human serum of patients with acute toxoplasmosis. It was added the peroxidase-conjugated anti-human IgM (DAKO, Denmark) diluted in 1% BSA-PBST20 (1/200 and 1/2000, respectively). An appropriate volume of the DAB (DAKO, Denmark) was poured on the paper and a brown band appeared. The band’s molecular weight was identified according to the protein marker. This band is not in non-transfected cells that confirmed the specific protein band.

## Results

### The results of pcDNA3 expression plasmid enzyme cut and pcGRA4 recombinant plasmid

**Fig. 1:**
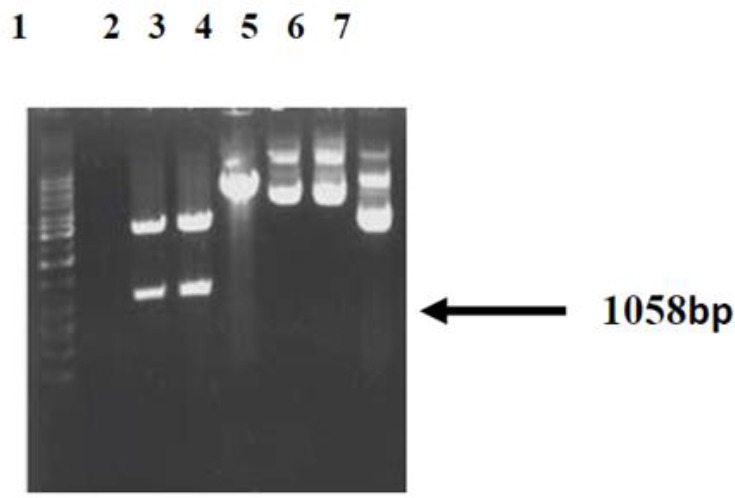
Restriction enzyme (KpnI and EcoRI) analysis of recombinant plasmid pcDNA3and pcGRA4. Lane 1: 1000 bpDNA ladder, Lanes 2–3: release of 1058 bp specific gene insert in pcGRA4, lane 4: pcDNA3, Lane 5–6: pcGRA4 without cutting, Lane 7 pcDNA3 without cutting

The pcGRA4 recombinant and pcDNA3 expression plasmids were cut with EcoRI and KpnI enzymes. The resulted pcGR4 recombinant enzyme plasmid which was cut (during concurrent enzyme cut reaction) with KpnI and EcoRI was electrophoresed and two bands with the weights of about 5. 4 Kbps (weight of pcDNAs without band) and about 1058bp (weight of GRA4) appeared. GRA4 gene was cloned in this plasmid (Fig. 1).

### Results of GRA4 PCR using pcGRA4 recombinant plasmid as atemplate

**Fig. 2:**
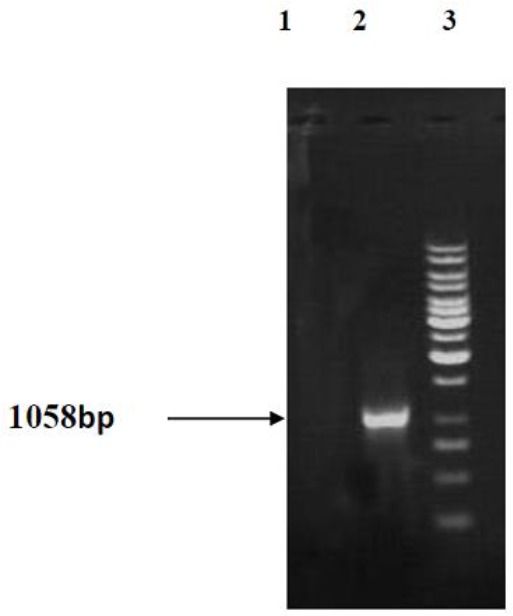
PCR amplification and gel electrophoresis. Lanes 1: pcDNA3, Lane 2: PCR product of pcGRA4 (approximately 1058 bp), Lane 3: 1000 bpDNA ladder

Results of PCR product electrophoresis with pcGRA recombinant plasmid using specific primers revealed that GRA4 gene’s 1058bp band has been amplified from pcGRA4 recombinant plasmid and the GRA4 gene, while there was no band in electrophoresis of the pcDNA3 plasmid’s PCR product. Therefore, cloning of GRA4 gene band in pcDNA3 plasmid was confirmed (Fig. 2).

### Results of pcGRA4 recombinant plasmid expression in CHO cells

In order to investigate GRA4 gene protein expression, the CHO eukaryote cells were applied. After transfecting CHO cells with pcGRA4 recombinant plasmid and the plasmid without GRA4 gene as control were cultured 72 h, the protein was collected, and was analyzed using SDS-PAGE and Westernblot methods.

### Result of determining the proteins molecular weight using SDS-PAGE

**Fig. 3:**
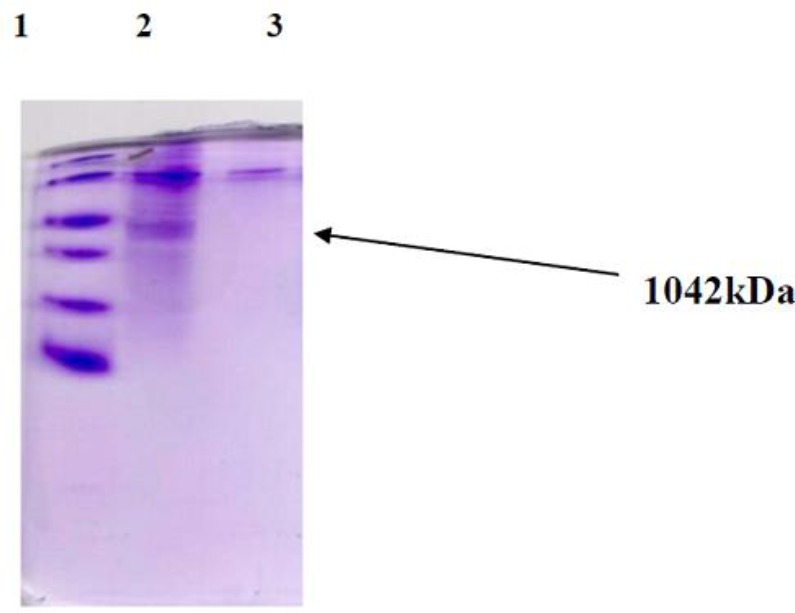
SDS-PAGE analysis on the expression of recombinant GRA4 in CHO cell. Lane 1: protein molecular weight marker (top to down 116, 66. 2, 45, 35, 25, 18. 4, 14. 4 kDa), Lane 2: contained supernatant ofCHO cells transformed, Lane 3: supernatant of untransformed CHO cells

The results of SDS-PAGE has revealed that the band in the column related to the cell well transfected by pcGRA4 plasmid have been observed in the weight area of about 42 kDa, while they are not in pcDNA3 plasmid column (Fig. 3).

### Result of Westernblot

**Fig. 4:**
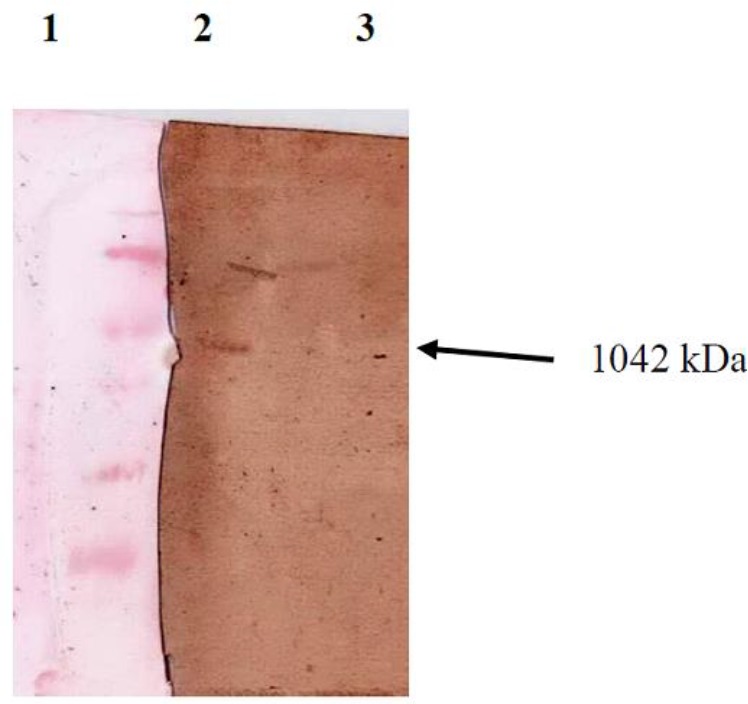
Western blotting showed human *T. gondii*positive sera recognizing GRA4 protein from transfected CHO cells. It was not detected in nontransfected control cells. Lane 1: protein molecular weight marker (top to down 116, 66. 2, 45, 35, 25, 18. 4, 14. 4 kDa), Lane 2: containingpcGRA4 plasmid with band at about 42 kDa, Lane 3: pc-DNA 3 (negative control)

The nitrocellulose paper on which the separated proteins are transferred fromthe SDS-PAGE gel, the molecular weight of protein band is about 42 kDa in the well transfected by pcGRA4 plasmid while there is no band in the control well. Formation of this band on the nitrocellulose paper shows that GRA4 protein is identified by IgM^+^ human anti toxoplasma serum (Fig. 4).

## Discussion

Observing the protection fetus from congenital infection during pregnancy in women chronically infected with *T. gondii* indicate the degree of immunity against *T. gondii*and in immunocompetent individual first infection result in protective immune response against second infection, suggest that effective vaccine can confer protection against this infection and congenital transmission(20). DNA vaccines have ability to stimulate CD4^+^ T-lymphocyte and CD8+ cytotoxic T-lymphocyte (CTL) responses against the antigen insert to expression plasmid (21). Oral infection with *T. gondii* induce humoral and cell-mediate immune responses by GRA4 peptides because amino acids 229–242 and 231–245 are epitopes for B and T-cell (16,17). Amino acids 229–242 from GRA4 induces noticeable proliferation of primed-CBA/J mice T lymphocytes (22). Serum IgG antibodies from infected sheep and humans, and IgA antibodies of milk and intestinal from infected mice recognized with Protein C (amino acids 297–345) of GRA4gene (23).

At first to for building a recombinant vaccine, we have to confirm the expression of GRA4 gene of *T. gondii* in eukaryotic cells (CHO). GRA4 gene cloning in pcDNA3 plasmid was confirmed using restriction enzyme and PCR methods. Then GRA4 gene expression was examined in vitro. Firstly, pcGRA4 recombinant plasmid was transfected in CHO eukaryote cells, using calcium phosphate method and with SDS-PAGE and Westernblot analyze the protein band with the molecular weight of about 42 kDa was distinguished. It was active because it could be recognized by human antibody positive serum from patient with acute toxoplasmosis infection. pET-32a expression vector was used to GRA4 gene expression in prokaryotic system and reported the gene’s molecular weight of about 50 kDa. The difference in the molecular weight is related to the histidine-tagged application (24). HEK293Teukaryotic cell was used to gene expression and found that the molecular weight was about 70 kDa, which composed of 40 kDa and 30 kDa weights of GRA4 gene expression and green fluorescent protein producer, respectively (25).

GRA4 gene was cloned into pPICZα A expression vector then integrated into the *Pichic. Pastoris* genome according to the manufacturer’s procedure of the Easy Select^TM^ *Pichia* Expression kit. The antigen expressed together with the pre-sequence of the α-factor of yeast. SDS-PAGE analysis confirmed that the recombinant protein expressed as a 40 kDa molecular weight. The antigenic reactivity found in the Western blot analysis (26).

Calcium phosphate transfection method was used base on Profection mammalian transfection system (Promega kit). Although they use Cos-7 cells for eukaryotic expression cell, the specific band with a molecular weight of about 40kDa were observed in western blot (15). GRA4 gene was cloned in pcDNA3 to produce recombinant eukaryotic expression vectorpcGRA4. Then performed transfection ofpcGRA4 in Cos-7 eukaryotic cells by using Lipofectamine method. The protein obtained was 40–41kDa molecular weight and was in the highest immunostimulatory effect (27). Therefore finding in this study approximate close to molecular weight of GRA4 protein expression in other studies. Calcium phosphate transfection is the method of choice to produce long-term stable transfectants. This method also works well for transient expression of transfected genes and can be used with most adherent cell lines.

## Conclusion

GRA4 gene that previous subcloned into pcDNA3 an expression plasmid can express protein in eukaryotic CHO cell. We will use from expression GRA4 plasmid to construct recombinant vaccines and evaluated the ability ofpGRA4 for protective immune response against toxoplasmosis in mouse models.
